# Rats: if you can’t beat them eat them! (Tricks of the trade observed among the Adi and other North-East Indian tribals)

**DOI:** 10.1186/s13002-015-0034-2

**Published:** 2015-05-30

**Authors:** Victor Benno Meyer-Rochow, Karsing Megu, Jharna Chakravorty

**Affiliations:** Research Institute of Luminous Organisms: Hachijo, 2749 Nakanogo (Hachijojima), Tokyo, Japan 100-1623; Department of Zoology, Rajiv Gandhi University, Doimukh, Arunachal Pradesh India 791111; Department of Biology, Oulu University, P.O. Box 3000, , FIN-90014 Oulu, Finland

**Keywords:** Adi and Apatani, Arunachal Pradesh, Food, Rat meat, Pest control, Minilivestock, Diet, Taboos

## Abstract

**Background:**

Since outside the tribal areas of North-East India it is not widely known, neither in the world nor in India itself, that rats are considered a delicious food item, this was one of several reasons why we decided to present this ethnographic account of rat procurement and preparation (together with some additional comments on the cultural role that rats have especially amongst members of the Adi tribe). Consumption of rats by humans as a biological control method far superior to the use of rodenticide poisoning and rat consumption as a way to reduce hunting pressure on rare wild animals were further considerations to publish this account.

**Methods:**

Semi-structured interviews were conducted with male and female members of eight tribal communities in Arunachal Pradesh (North-East India) on the uses of rats as food and as cultural objects. The construction of rat traps as well as the preparation of rat dishes were observed and recorded photographically.

**Results:**

Numerous species of small rodents, collectively called “rats” by the locals of North-East Indian tribes and comprising the species *Rattus rattus* Linnaeus*, R. nitidus* Hodgson*, R. burrus* Miller*, R. tanezumi* Temminck as well as *Bandicota bengalensis* Gray and Hardwicke*, B. indica* Bechstein*,* and *Mus musculus* Linnaeus, are regularly trapped and consumed in roasted, cooked or smoked form. In this well-illustrated report the kinds of devices used to catch these animals are described and information is provided on how to prepare rats for human consumption. The role that rats as food and gift-exchange items play in the context of local culture is explained and the locals’ most highly appreciated meat dish, known as *bule-bulak oying* and consisting of boiled rat’s tail, legs and inner organs, is introduced.

**Conclusion:**

Given the need to meet the world’s future food demands and the environmental consequences of an expanding livestock production with regard to global warming, water availability, deforestation, soil erosion etc., rats as a food item, as our example shows, should not be overlooked. Using rats as food reduces hunting pressures on other wild and often already rare animals. It is a far superior method to control rat populations than poisoning the rodents and the artisanal construction of rat traps by local menfolk helps maintaining traditional skills and knowledge.

## Introduction

Apart from a few individuals, who keep rats as pets and some scientists who carry out experiments on them, rats are genuinely disliked by people with a European cultural background [1]. Rats are seen as dirty, sneaky little creatures that enter houses to steal food at night and transmit diseases (e.g., bubonic plague comes to mind: [2]). The age-old struggle between rats and humans in Europe has found its reflection in numerous fairy tales, stories and even derogatory idioms.

**Fig. 1:**
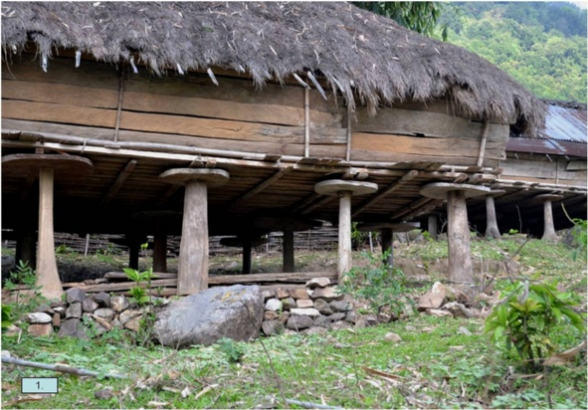
Large wooden discs that rats cannot pass are placed between the storage house and the pillars it rests on

In many Asian countries the attitude towards rats is a different one. There, too, people are of course not ignorant to the damage rats can cause to their stored food products and people of the Adi tribe, for instance, construct store houses in such a way that rats cannot easily get into them (Fig. 1). A variety of traditional rat-proofing designs have been reported [3]. However, despite wanting to keep rats out of the storage areas, credit is given to the rats’ intelligence, their adaptability and hardiness and even temples (e.g., the Karni Mata) dedicated to rats exist in India, because Hindus believe that a rat had helped to carry Lord Ganesh around the world [4].

**Fig. 2:**
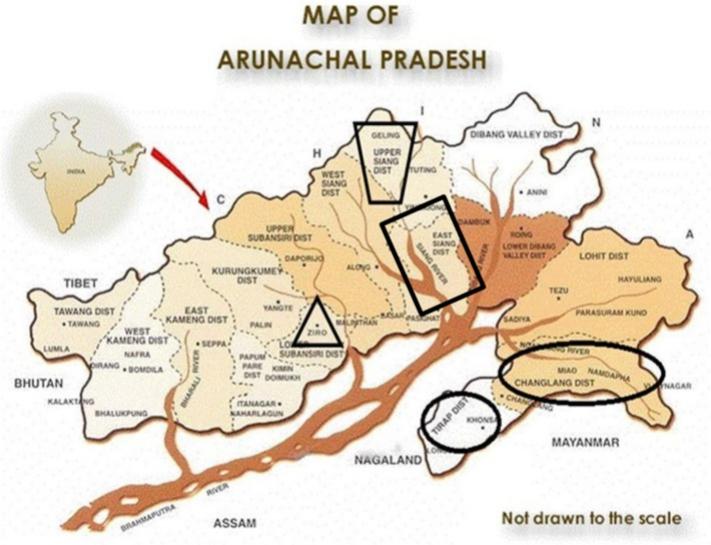
Map of North-East-India, showing areas inhabited by rat-eating tribes visited by us. Triangle: Apatani; trapezoid and rectangle: Adi; long oval: Tangsa, Singpho, and Tutsa small oval: Wangcho, Nocte, and Tutsa

In tribal areas of North-East India (Fig. 2) amongst the various ethnic groups of Nagaland and Arunachal Pradesh (Thakur et al [5] mention tribal communities “numbering in excess of 50”) rats are appreciated for yet another reason: their taste. Since outside the tribal areas it is not widely known, neither in the world nor in India itself, that rats are eaten by humans, this is one of several reasons why we decided to present this ethnographic account of rat procurement and preparation (together with some additional comments on the cultural role that rats have especially amongst the Adi).

There are, however, several extra reasons why we feel our ethnozoological observations would be useful. The first relates to the sustainability of an age-old lifestyle practiced by the tribals of the region in question. The latter has repeatedly been referred to as a “biodiversity hotspot” [6, 7], but species diversity is under threat from increased hunting activities by local hunters and from the nowadays widespread use of firearms in preference to traditional methods [8]. Populations of bigger species like wild elephants, tigers, bears, wild boars and even some smaller animals like pangolins as well as numerous species of birds of the region, irrespective of whether legally protected or not, have already declined dramatically, which is alarming since the North-East’s Arunachal Pradesh, with 80 % of the land area covered in forests, is considered home to 65 % of the total number of mammalian species in India [9].

Given, however, the high appreciation of rats as a meat source in tribal North-East India (this paper), the widespread use of rats as food can only be welcomed and encouraged since rats themselves are not (yet) an endangered species and their non-use would create an even more serious pressure than what it is currently on the remaining wildlife. Moreover, since rats are a threat to stored food products like grain, tubers and other edibles, eating the rats seems more sensible than only killing and not using, or worse, poisoning them and leaving their corpses to be devoured by other organisms. An additional bonus of using rats as food is the fact that in order to catch them, traditional traps constructed of natural and locally available materials are employed and skills to manufacture and set the traps are not lost.

There is one more important aspect that prompted us to publicize the rat-eating habit of the tribes of North-East India: the global situation of future food security. Although there is evidence that hominoid dietary evolution began with fruitivory [10], meat in the human diet historically became so important [11] that today it is widely debated how the demand for more meat-containing products can be met in the future [12–14]. Environmental consequences of an expanding livestock production are being discussed with regard to global warming, water availability, deforestation, soil erosion etc. affecting the entire world [15–18] and specifically developing countries including India [19, 20]. Although it has repeatedly at least since 1986 [21] been suggested that non-traditional meat animals like rodents [22–25] could have a role to play in meeting future global food (and in particular protein) security, the aversion to accept rats as food is deeply rooted. To show that this need not be so, is yet another reason for this report, in which we present tribal food practices as an example that other cultures can learn from.

Because of a total lack of a zoo-archaeological record of rat consumption in North-East-India, it is not possible to state exactly since when rats have been part of the diet of the local people. It is, however, certain that it was not a response to an increasing population or lack of other food items that made them accept rats as food and that catching and eating rats has a long history in the region. Although these days some of the bigger wild animals like elephants, tigers, bears, wild boars etc. have indeed become very rare, there is still no shortage of many larger edible wild animals like buffalo, barking and musk deer, mountain goat, porcupine or numerous species of birds and reptiles, to name but a few. And yet, according to the locals “nothing beats the rat”.

A delicious meal, a treat for the family and friends: for such occasions and festivals like the traditional “*unying-aran*” of the Adi people, or for the unexpected arrival of a friend, a celebration amongst the Apatani rats are a *must* on the menu. To learn that for a member of the Adi tribe or an Apatani family the most delicious parts of a rat are its tail and legs may make many a person of European cultural background cringe, because to them rats are vermin, they are taboo, but for tribal North-East Indians just to mention these food items makes their mouth water. The situation, in fact, is not unlike certain food taboos discussed in Meyer-Rochow [26] that operate in one society, but do not exist in another. However, before one can indulge in such delicacies as rat tails or rat stew, one needs to catch and prepare the rats and to describe how this is done in North-East India, together with a serious attempt to convince a wider public of the value rats and other rodents represent as food items, is the focus of our report.

## Material and methods

Inquiries into the uses of rats amongst tribes of Eastern Arunachal Pradesh namely Nocte, Wangcho (Wancho), Singpho, Tangsa, Deori and Chakma were carried out in 2012 during the months of March (2 weeks) and April/May (10 days) after on numerous occasions rats were seen to be prepared for the evening meal. The number of households per village was 20–25. At least two to three households inhabited by village elders and their families were visited. Recommendations by the headman or village elders to interview certain knowledgeable persons were sometimes followed. The surveys were based on interviews during which a total of 20 persons (males as well as females aged between 45 and 70 years of age) were present. However, since the main emphasis of the field research amongst these tribes had been research into entomophagy and entomotherapeutic uses of insects [27], only a few simple questions as to whether rats were used as food and regarded as delicious or represented perhaps an item that one would accept only if nothing better was available, had accompanied the questions primarily related to insect uses at that time.

Interviews specifically aimed at elucidating the roles that rats and related rodents play as an item of food and cultural importance were conducted amongst members of the Adi and Apatani tribes. Interviews were held in the local language (one of this paper’s authors, K. Megu, is a native Adi man), and occasionally in Hindi or English or with the assistance of an interpreter. In thirty households out of a total of 110 in the Adi village of ‘Motum’ gatherings were arranged mainly during the months of February – April 2014 and December 2014 - March 2015 in which all family members participated in contributing information on the uses and roles of rats. Answers were copied down on paper and later fed into a computer back at Rajiv Gandhi University (Doimukh, Arunachal Pradesh).

**Fig. 3:**
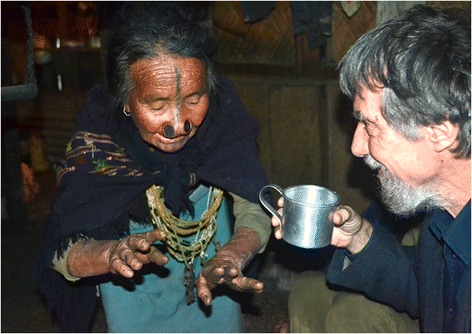
An old Apatani lady explains to senior author Meyer Rochow how to cook rats

Interviews with members of the Apatani tribe (Fig. 3) in the settlements Hong, Hari, Hija, Bulla, Duta, Bamin, Michi around the town of Ziro (located in the valley of Subansiri District; Arunachal Pradesh) followed a similar practice and involved sessions with four families (with up to 7 and at least three members present). Additional interviews, however, took place and involved a local school teacher, a local guesthouse manager and small groups of Apatani farmers and government agricultural advisors in Ziro. When practical, it was requested that we be shown how rat traps were manufactured, how they were set, and how the rats were prepared for human consumption. Visits to the Apatani region took place during the month of October 2014. The research was approved by the Ethics Committee of Rajiv Gandhi University and consent was obtained from all informants to record and publish their responses and any accompanying images. Names of the informants were not kept unless the informants agreed to having had their names written down.

Adi and Apatani, tribal communities (according to the 2011 census by the Government of India) number approximately 150,000 and 28,000 members, respectively, and lead largely traditional lives based on agricultural activities and hunting or trapping of wild animals. In fact, one of the authors of this paper, K. Megu, is an Adi man, raised in the Adi village of Motum in the East Siang District, and now a PhD.-student of Ethnobiology at Rajiv Gandhi University. The other two authors, V.B. Meyer-Rochow and J. Chakravorty, together with students, have made numerous field trips over the years from 1990 until 2014 to various places in North-East India to study food habits of tribal communities in collaboration with local student and scientists [27–29].

This report represents “a descriptive, ethnographic account”; statistical analyses were not required. Although the details given on the customary use of rats are those of the Adi tribe, those dealing with the preparation and appreciation of rats as a component of the local diet are identical amongst the Apatani and, indeed, all the tribes of the region that were contacted by us on field trips over the last few years to determine the extent of rat-consuming practices.

## Results

**Fig. 4:**
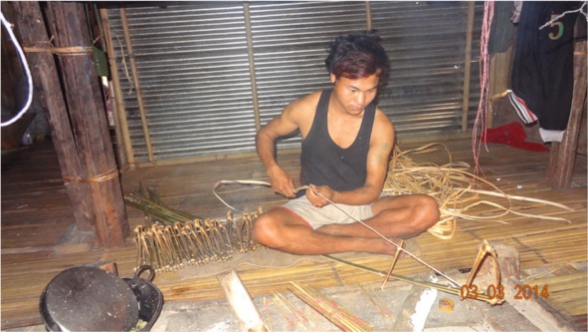
How to construct traps out of bamboo and string to catch rats is learned by boys at home from elders

**Fig. 5:**
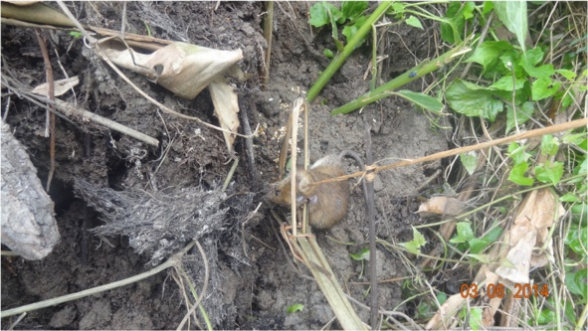
Rats snared in traps that are set in the field and bush around the house, die by asphyxiation

Throughout the rat-consuming tribes, simple, but sophisticated designs of traps exist that are made out of locally available plant materials like bamboo and string, and very rarely wire [5, 30]. Young Adi boys from the age of 7 learn from the older menfolk how to construct such traps (Fig. 4). The traps are set in areas of the bush and fields around the houses, in places known to be frequented by rats. Only rats caught in the countryside from the forest or agricultural lands outside the locals’ dwellings are considered acceptable as food (although exceptions are made, when the desire for a meal of fresh rat meat is simply too great). The rats − and for the locals any rodent of rat-size is a rat, whether it is *Rattus rattus* Linnaeus*, R. nitidus* Hodgson*, R. burrus* Miller*, R. tanezumi* Temminck or any other species, including *Bandicota bengalensis* Gray and Hardwicke*, B. indica* Bechstein*,* or *Mus musculus* Linnaeus as well as possibly undescribed species − usually get snared behind the head (Fig. 5) and either die through asphyxiation or will be killed when the successful traps are collected. The traps are non-selective and rats are caught indiscriminately.

Adi tribals employ four kinds of strategies to catch rats. The most common type of trap is called “*etku*” and is made out of just two materials: bamboo for the main frame and *sargok* string (obtained from the bark of a tree and also used to tie up bigger animals like cows, mithun [*Bos frontalis*], buffalo, etc.) for ensnaring the prey. At the end of the bamboo bow a small triangle of bamboo strips is an essential part of the trap. At the other end of the bow a string is fixed to a wooden spike. The trap is set, when tension is provided to the bow by fixing the wooden spike ever so lightly inside the triangle that contains a noose, on which some bait is deposited. When a rodents (or a bird), attracted to the bait (which consists of a paste of stink bug *Aspongpopus nepalensis* with dough of local rice) or passing through the noose, the weight of the prey presses the wooden spike and releases the bow’s tension and strangles the rat or bird or even a snake – whatever small animal got between the string in the triangle. The *etku* trap is very effective and also used inside houses.

The second type involves a bamboo smoke pipe and is known as “*medung*”. A mixture of burning charcoal and husk is put inside the bamboo to produce smoke. The smoke is then blown into the rat burrow and makes the rat or rats come out lest they die of smoke suffocation. Although also effective, this method of catching rats is now much less frequently used, because of the danger associated with it to set dwellings ablaze.

The “*songkit*” is a type of trap that can be used in catching rats or some other animals, e.g. snake, bird, lizard. A small noose of a rope, worked through a system of levers, is placed at the mouth of the animal’s burrow (or the track the animal uses). The lever, e.g. a bent branch or a manually placed flexible stick, to which the spike along with the noose is attached is released by the prey and shoots up in the air with a jerk, strangling the animal to death.

The method known by the name of “*eda*” was commonly employed in the past, but abandoned more recently probably due to its lesser efficiency or because its operation involved considerable expertise and/or harder manual work. A heavy material (commonly a stone) was supported by wooden posts under which the bait was placed. A rat entering the contraption to steal the bait could cause the wooden posts to topple and the loosely supported, heavy stone to fall onto the rat and kill it. This technique used to be widely accepted and the method of choice by specifically Abotani clans, Adi, Apatani, Nyishi, Galo, and Tagin.

Depending on the number of traps set, on a good day, defined by the catch, some 30 (and even up to 100) rats may be caught by a single rat-hunter. Usually traps are set in daytime, left overnight, and visited the next morning to remove any rats caught in them and to check if traps got damaged or caught unwanted animals. Sometimes it takes only a few hours before the first rats are caught. The number of rats removed from the wild by all the hunters of a community in this way is staggering and ample proof of the rat’s abundance and fertility. In the village Motum, for example, as a conservative estimate 150 active hunters may catch at least 50 rats each (=7,500 rats) during the festival month alone. The biological control, exerted through the human consumption of the rats, keeps rat populations at a relatively constant level and is far superior environmentally to the use of chemical rodenticides, many of which are now known to seriously affect humans as well [31]. There have been warnings, based on precedences, as to what can happen if rat populations grow unchecked by human collecting pressure, especially during times of gregarious bamboo flowering and seed production that create ideal conditions for a rat population explosion [32].

When an Adi or Apatani man returns home with a day’s catch of rats (and it is always the men who build, set and control the traps, because traditionally women’s activities revolve around garden and house work) there is great joy amongst the village children and of course the hunters’ wives, who, not being involved in catching rats, may have instead collected wild edible vegetables in preparation for the rat dish. Although available and consumed throughout the year, rats are best, because they are then fat, during the time of “*pime*” (the harvest festival), which, depending on subtribe and village, occurs from November to February. The biggest festival in the region is the Adi’s “*unying-aran*” hunting festival on March 7 th every year. For that festival enormous numbers of rats, in fact thousands, and some other animals, mostly wild birds, are procured. During the summer months rats are lean and not nearly as tasty as during the winter. Recently trapping rats for commercial purposes has begun and rats are sold at local markets, because serving the meat of a rat is regarded as more prestigious than serving meat of larger vertebrates.

The first morning of the *unying-aran* festival is known as the “*aman ro*” (the morning of gift giving), on which the hunters present their catches to their families. Children of every household are given a pair of dead rats similar to the practice of giving toys to children during Christmas (Arunachal tribals are mostly Christians or followers of the Donyi Polo religion [33]). The practice of “gift-exchange” is also widely practiced and amongst the Adi each family gives a pair of rats to their real or closely related both maternal and paternal uncles, while uncles reciprocate with local wine. Hence, the use of rats as gifts strengthens relationships in the Adi Society.

Rats also play a role in the Adi’s matrimonial system. According to tradition the bridegroom’s family will not recognize the bride unless a ceremony has taken place, in which five pairs of smoked, dried rats along with some meat of squirrels and larger animals (mithun, buffalo, pig, barking and musk deer, etc.) are presented to the bride’s family as a tribute, which is accepted and leads to an offering of local wine in return. Questioned by us about the origins of this custom, we drew a blank and there is seemingly no knowledge of a connection to local mythology or spiritual mandate as with the Hindu and their appreciation of the rat [4]. There is also no evidence that the rat serves as a substitute for something else now extirpated or rare.

**Fig. 6:**
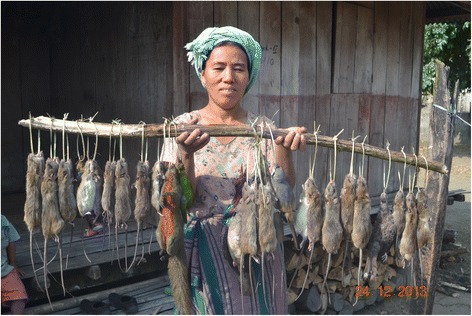
Freshly caught rats are hung on racks under the roof of the house

**Fig. 7:**
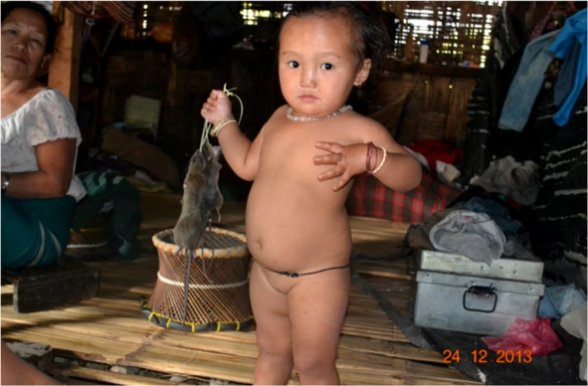
Even the smallest already experience the joy of catching (or at least holding) rats

**Fig. 8:**
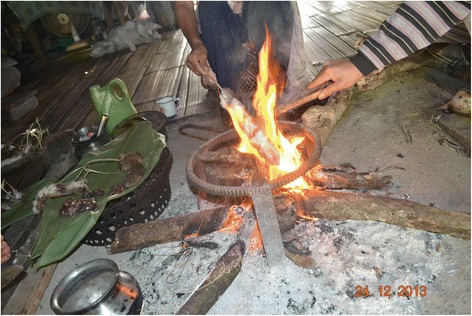
Rats on a stick are roasted whole over an open fire

**Fig. 9:**
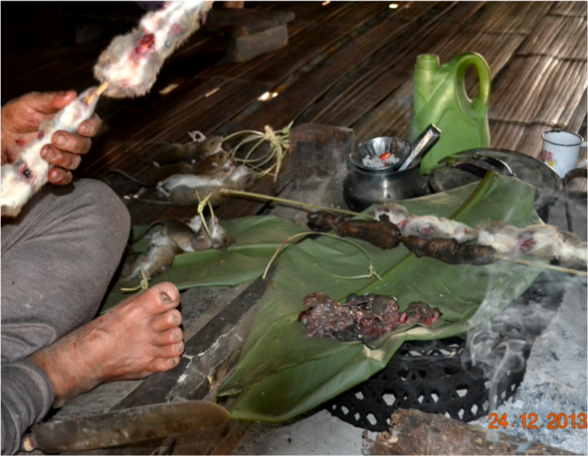
Often, but not always, the inner organs of the rat for use in a stew are first removed before the gutted rat is roasted

The dead rats are hung up under the roof (Fig. 6), but those attached to strings and given to children are proudly carried around by them (Fig. 7). Dead rats can keep a while and need not be gutted and consumed straightaway. Some elderly folk even prefer rats in the process of decay with maggots in them and wait until the smell tells them it is time to consume rat and maggots together. However, to preserve the rats for longer periods as an emergency or to feed an unexpected guest, they are usually roasted or smoked with tails and legs cut off and turned into a stew as quickly as possible. The fur is simply singed off over the fire and internal organs are sometimes, but not always removed (Figs. 8 and 9).

**Fig. 10:**
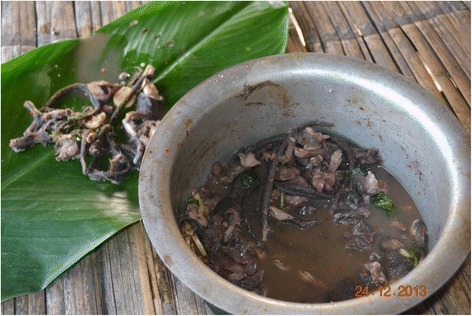
Considered most tasty, legs, tails and inner organs of the rat are separated to be stewed together

In case a rat is gutted, only the gizzard and the end gut containing unexpelled faeces, are removed. Internal organs like, e.g., stomach, intestines, liver, testes, foetuses, etc. are put aside and then boiled together with tails and legs as well as some salt, chili and ginger (Fig. 10). This dish is known locally as “*bule-bulak oying*” and widely appreciated as the most delicious of all rat-based foods. Although food taboos exist, which preclude the consumption of stick insect, praying mantis and vultures as agents of evil, none are associated with eating rats or their parts so that all members of a family, irrespective of age and gender, can partake in the meal. However, at no time are any parts of a rat ever eaten raw. Almost the only part of a rat not used are its teeth; even the rat’s bones are consumed. If a person gets angry easily, one accuses that person jokingly of having eaten the rat’s head.

**Fig. 11:**
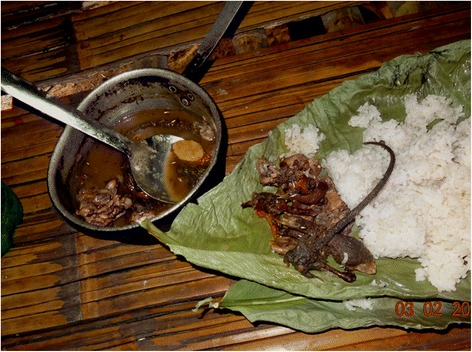
The meal is served on a leaf and eaten with the fingers

The boiled meaty parts are either consumed on their own or with a helping of sticky rice and some green leafy vegetables. Rats wrapped in leaves of the palm *Livistona jenkinsiana* Griff, known locally as “*toko patta*”, can also be roasted over fire. Knives and forks are not used, because the full appreciation of the food is only gained when in addition to the visual delight, the smell and the taste of the food its texture can be felt as well (Fig. 11). Meals are served on *toko patta* leaves or occasionally banana leaves, an item that is easily re-cyclable and therefore preferable to plates made of plastic and even porcelain, the latter needing to be washed and cleaned with soap.

## Discussion and conclusions

Use of rats as a human food item is far more widely common than generally appreciated by Westerners, but in the past has been documented most frequently for African and South American communities [34–39]. However, tribal communities in all North-Eastern states of India contain rat-consuming communities and an appreciation of rat meat is further present amongst tribals of the adjacent countries of China and Myanmar (S. Changkija of Nagaland University, pers. comm.) as well as the rest of South-East Asia. Bush rats are considered to be clean animals and according to members of rat-eating communities their meat is said to be tasty and healthy. The recently reported risk of acquiring *Leptospirosis* via direct contact with infected rats, their tissue and urine [40] in Southeast Asia is either very low in the tribal areas of Northeast India or is ignored by the locals.

Given the value the rat has in the various rat-consuming ethnic groups of Northeast-India, the conservation of this resource through rat-trapping as a means of sustainable management can be expected, but we cannot provide any evidence that conservation is actively enforced through perhaps a seasonal hunting ban or by limiting the catch per hunter. The avoidance of toxic chemicals (like alumi-nium phosphide) for rodent control purposes reduces environmental contamination generally and pesticide-related health problems to humans in particular [31]. Trapping wild rats in the way the Adi, Apatani and others are practicing it, is an effective biological control method and for this reason preferable (not just for Northeast India, but other regions as well) to the occasionally aired view of farming rats.

Although the widespread use of pesticides has been called into question on account of its side-effects on human health and in South Africa rat traps have been found to be acceptable to “poor urban communities as an alternative to toxic pesticides” by Roomaney *et al* [41], these authors have not considered any possible uses of the trapped rodents (be it as food or otherwise) even if elsewhere in many African countries these animals represent an accepted food item. Whether the thought that “if you can’t beat them, eat them” never occurred to them or they were hesitant to express such an idea we do not know.

That rodents can be a suitable source of meat even for people generally, irrespective of their cultural backgrounds, has been pointed out by Fiedler [36] and given the need to, on the one hand, increase food production by 70 % in order to “feed the world in 2050” [42] and on the other to drastically reduce ruminant meat consumption to avoid further global warming [18], earlier calls for rats as “minilivestock” [21–23] to be farmed in the future [24, 25] now have to be taken seriously.

In terms of the potential as a food item, already in 1996 Zorzi and Chardonnet [43] had been able to show that rodents accounted for 40.4 % of the animal species consumed in the Upper Congo; a value far exceeding that of artiodactyls (28.5 %), primates (19.1 %) and others. Dividing the quantity (gram per capita eaten per day) of rodent meat consumed by the total quantity of game meat gave ratios of 18.3 % in Nigeria, and, depending on the area, 5.5 – 25 % in Gabon, 20-48.8 % in Togo and 31.5 – 35 % in the Côte d’Ivoire [44]. Although unavailable for Northeast Indian rat-consuming tribal communities, similar ratios can be expected, but may vary seasonally.

The question is, given the long standing apprehension of anything rat-related by people with European cultural backgrounds, will European or North American gourmets ever accept rat on the menu? This is difficult to predict, because forty years ago many food specialists did not think that raw fish would ever become acceptable to Europeans (“we are not cats” was a comment then heard frequently). But ‘sushi’ has now become an item available in even the smallest European villages and insects as human food, according to some scientists’ predictions, are poised to become the next new culinary item to be embraced by Europeans. But rats? If prepared in a way that does not make it obvious that one deals with rat meat (fish sticks come to mind, or sausages, or the various kinds of *paté* that do not betray the species that they were made from), then there could be a chance of a change in attitude. However, for tribal people of North-East India at least “no rat, no feast, no celebration” applies.

A personal remark to end this report with may be in order: having tasted fried rat leg, the senior author of this paper (normally being a vegetarian unless tasting meat is part of his research), did not find the taste much different from that of other kinds of meat he had tasted before. However, he found that the rat’s characteristic smell identical to that of laboratory rats was lingering on even in the meat of the leg of the fried rat he was offered to taste. Based on that experience, he’d conclude that the acceptance of rat as a food item in European-dominated societies is likely to be a long and tough uphill struggle.
